# Independent replication analysis of genetic loci with previous evidence of association with juvenile idiopathic arthritis

**DOI:** 10.1186/1546-0096-11-12

**Published:** 2013-03-18

**Authors:** Justine A Ellis, Raul A Chavez, Angela Pezic, Anne-Louise Ponsonby, Jonathan D Akikusa, Roger C Allen, Jane E Munro

**Affiliations:** 1 Genes, Environment & Complex Disease, Murdoch Childrens Research Institute, 50 Flemington Rd, Parkville, Vic, 3052, Australia; 2 Environmental & Genetic Epidemiology Research, Murdoch Childrens Research Institute, Parkville, Vic, 3052, Australia; 3 Arthritis & Rheumatology, Murdoch Childrens Research Institute, Parkville, Vic, 3052, Australia; 4 Rheumatology Unit, Department of General Medicine, Royal Children’s Hospital, Parkville, Vic, 3052, Australia

**Keywords:** Juvenile idiopathic arthritis, Genetic association, Independent replication

## Abstract

**Background:**

Over the last five years, there have been numerous reports of association of juvenile idiopathic arthritis with single nucleotide polymorphisms (SNPs) at various loci outside the major histocompatibility complex (MHC) region. However, the majority of these association findings have been generated using a limited number of international cohorts, and thus there is benefit in further independent replication. To address this, we examined a total of 56 SNPs in 42 non-MHC gene regions previously reported to be associated with JIA, in the ChiLdhood Arthritis Risk factor Identification sTudY (CLARITY), a new Australian collection of cases and healthy child controls.

**Findings:**

Genotyping was performed on a total of 324 JIA cases (mean age 9.7 years, 67.3% female) and 568 controls (mean age 7.8 years, 40.7% female). We demonstrated clear evidence for replication of association of JIA with SNPs in or around *c12orf30*, *c3orf1*, *PTPN22*, *STAT4*, and *TRAF1-C5,* confirming the involvement of these loci in disease risk. Further, we generated evidence supportive of replication of association of JIA with loci containing *AFF3*, *CD226*, *MBL2*, *PSTPIP1*, and *RANTES (CCL5).* These results were robust to sensitivity analyses for ethnicity.

**Conclusion:**

We have provided valuable independent data as to the underlying genetic architecture of this understudied pediatric autoimmune disease, further confirming five loci outside the MHC, and supporting a role for a further five loci in determining disease risk.

## Findings

Our understanding of the genetic basis of juvenile idiopathic arthritis (JIA) has recently increased, but still lags behind many other autoimmune diseases. This is largely due to the paucity of DNA collections internationally. While association of JIA with variation in the major histocompatibility complex (MHC) is well-established [1], over the last five years, there have been a number of reports of new JIA susceptibility loci that lie outside this region. These findings have resulted from candidate gene approaches (for example, examining genes known to be associated with rheumatoid arthritis), and more recently, from a limited number of genome-wide association study (GWAS) approaches. The vast majority of reports describe discovery and replication findings generated from two large sample collections, from the UK and the US [2]. Thus, although solid evidence for association with JIA has usually been described, for many of the identified loci, further replication in an entirely independent JIA sample would be beneficial in confirming their contribution to disease risk.

To address this, we performed a single nucleotide polymorphism (SNP) replication study within a new sample of JIA cases and healthy hospital-based child controls, collected so far as part of the ongoing Australian CLARITY study (ChiLdhood Arthritis Risk factor Identification sTudY) [3]. We selected 56 SNPs from 42 gene regions. Candidacy was based on published evidence of association, or a trend towards association, with total JIA, or one or more of its subtypes. These genes included *ADAD1-IL2-IL21*[4-6], *AFF3*[5], *ANGPT1*[6], *ATXN2*[7], *BACH2*[7], *c12orf30*[6,8], *c3orf1*[9], *CCR5*[10], *CD14*[11], *CD226*[5], *CD247*[2], *CLEC16A*[12], *COG6*[6], *CTLA4*[5], *ERAP1*[13], *IL12A*[7], *IL15*[9], *IL23R*[6,13], *IL2RA*[2,6,14], *IL7R*[5], *JMJD1C-REEP3*[9], *KIF5A*[15], *LPP*[7], *MBL2*[16], *MEFV*[17], *NLRP3*[17], *NOD2*[17], *NRBF2-EGR2*[9], *PRKCQ*[15], *PSTPIP1*[17], *PTPN2*[2,6], *PTPN22*[6,18], *RANTES (CCL5)*[19], *STAT4*[6,8,15], *TNFA*[20], *TNFAIP3*[8,15], *TRAF1-C5*[15,21], and *VTCN1*[22]. Additional SNPs from four other loci not attributed to any gene in the original publication but lying closest to the genes *DCN1*, *FHIT*, *HUNK*, and *SLITRK5*[22] were also selected. Genotyping was performed on a total of 324 JIA cases (mean age 9.7 years, 67.3% female) and 568 controls (mean age 7.8 years, 40.7% female) using the Sequenom MassARRAY system (assay design details available from the authors). After quality control pruning (removal of SNPs with Hardy Weinberg Equilibrium p < 0.01, and SNPs or samples with < 90% genotyping success rate) genotypes were successfully generated for 51 SNPs at 38 gene loci in 318 cases and 556 controls. SNPs excluded from analysis are listed in Additional file 1. Allelic, genotypic, additive (Cochrane-Armitage test for trend), dominant and recessive association analyses were performed using PLINK v1.07 [23]. Given the *a priori* evidence for association of each SNP, no adjustments for multiple testing were made.

**Figure 1:**
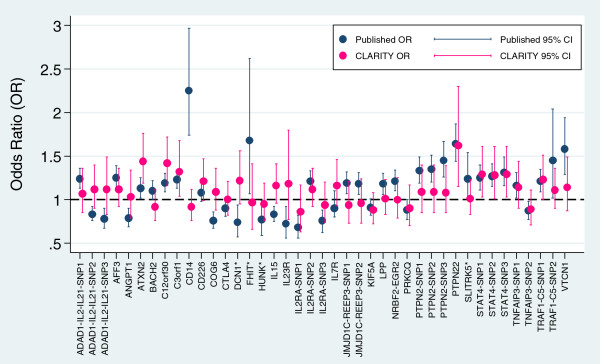
**Forest Plot comparing previously published odds ratios (ORs) with CLARITY ORs for genes examined via nearby SNPs.** Where more than one previously published OR was identified for a SNP, the OR based on the largest sample size was used. Some examined genes were excluded from this figure (see text for explanation). For genes with multiple SNPs (eg ADAD1-IL2-IL21) the SNPs are presented in the same order as listed in Table 1. * No gene attribution in original publication, closest gene by UCSC Genome Browser listed.

Outcomes of the association analyses in CLARITY cases and controls, including p values for the most highly significant test and allelic test for all SNPs analysed is shown in Table 1. Figure 1 shows a forest plot of the allelic odds ratios generated in the CLARITY sample compared to previously published odds ratios (ORs) for the majority of SNPs tested. Some SNPs were not included in this figure, including SNPs for which an OR was not presented in the original report, or where SNPs were previously associated specifically with rarer JIA subtypes. Our data demonstrates clear evidence of replication (defined as p < 0.05 for any test, ORs or case-control allele frequency differences in a direction consistent with previous reports) for SNPs at loci containing the genes *ATXN2*, *c12orf30*, *c3orf1*, *PTPN22*, *STAT4*, and *TRAF1-C5*. Possible evidence of replication (defined as p < 0.2 for any test, ORs or case-control allele frequency differences in a direction consistent with previous reports) was also generated for SNPs near *AFF3*, *CD226*, *MBL2*, *PSTPIP1*, and *RANTES (CCL5).* For *IL15*, we found a significant association, but in the opposite direction to that previously reported. The *IL15* SNP is an A/T transversion with frequencies of both alleles close to 50%, and thus there is the possibility of allele reversal. Although our minor T allele frequency in controls was entirely consistent with that found in the prior study, it is difficult to be sure that the *IL15* assays used in both studies were based on the same DNA strand. We therefore cannot be sure if our data supports replication. Additionally for *CTLA4*, considered a general autoimmunity susceptibility gene but with conflicting association results for JIA [5,24], we did not generate any evidence of replication.

**Table 1:** Clarity SNP association results, full sample

					**Best test p**^**§**^	**Allelic**		**Evidence for replication?**^**‡**^
**Gene**	**SNP**	**Minor allele**	**Case MAF**	**Control MAF**		**P**	**OR (95% CI)**	
ADAD1-IL2-IL21	rs17388568 [6]	A	0.25	0.24	0.29 R	0.55	1.07 (0.85, 1.36)	N
	rs13143866 [6]	A	0.28	0.26	0.28 D	0.31	1.12 (0.90, 1.40)	N
	rs6822844 [4,5]	T	0.13	0.12	0.42 R	0.46	1.12 (0.83, 1.49)	N
AFF3	rs1160542 [5]	G	0.49	0.46	0.16 R	0.25	1.12 (0.92, 1.36)	P
ANGPT1	rs1010824 [6]	T	0.17	0.16	0.71 D	0.82	1.03 (0.79, 1.34)	N
ATXN2	rs653178 [7]	G	0.55	0.46	0.00023 A	0.00023	1.44 (1.19, 1.76)	Y
BACH2	rs11755527 [7]	G	0.44	0.46	0.34 D	0.41	0.92 (0.76, 1.12)	N
C12orf30	rs17696736 [6,8]	G	0.50	0.41	0.00044 T	0.0005	1.42 (1.16, 1.72)	Y
C3orf1	rs4688011 [9]	A	0.24	0.19	0.016 D	0.02	1.32 (1.04, 1.68)	Y
CD14	rs2569190 [11]	A	0.48	0.50	0.24 R	0.43	0.92 (0.76, 1.12)	N
CD226	rs763361 [5]	T	0.52	0.48	0.059 A	0.059	1.21 (0.99, 1.47)	P
CLEC16A	rs6498169 [12]	G	0.37	0.35	0.30 A	0.3	1.11 (0.91, 1.36)	N
COG6	rs7993214 [6]	T	0.30	0.29	0.41 T	0.41	1.09 (0.88, 1.36)	N
CTLA4	rs3087243 [5]	A	0.46	0.46	0.80 R	0.97	0.997 (0.82, 1.21)	N
DCN1*	rs939898 [22]	G	0.21	0.18	0.057 D	0.11	1.22 (0.95, 1.56)	N
ERAP1†	rs30187 [13]	T	0.39	0.37	0.36 D	0.46	1.08 (0.88, 1.32)	N
FHIT*	rs9311745 [22]	C	0.07	0.08	0.86 A	0.86	0.97 (0.66, 1.41)	N
HUNK*	rs2833547 [22]	T	0.25	0.26	0.46 D	0.66	0.95 (0.76, 1.19)	N
IL15	rs13139573 [9]	T	0.47	0.43	0.0016 G	0.13	1.16 (0.96, 1.41)	?
IL23R	rs11209026 [13]	A	0.07	0.05	0.22 T	0.23	1.29 (0.85, 1.94)	N
	rs11465804 [6]	G	0.06	0.05	0.44 T	0.45	1.18 (0.77, 1.80	N
IL2RA	rs12251307 [6]	T	0.11	0.12	0.34 A	0.34	0.86 (0.63, 1.17)	N
	rs706778 [2]	A	0.45	0.43	0.26 T	0.26	1.12 (0.92, 1.36)	N
	rs2104286 [6,14]	G	0.21	0.22	0.21 R	0.63	0.94 (0.74, 1.20)	N
IL7R	rs6897932 [5]	T	0.25	0.23	0.17 D	0.19	1.16 (0.93, 1.46)	N
JMJD1C-REEP3	rs6479891 [9]	T	0.17	0.18	0.44 D	0.64	0.94 (0.73, 1.21)	N
	rs12411988 [9]	C	0.16	0.17	0.47 R	0.74	0.96 (0.73, 1.24)	N
KIF5A	rs1678542 [15]	C	0.36	0.39	0.22 A	0.22	0.88 (0.72, 1.08)	N
LPP	rs1464510 [7]	T	0.46	0.46	0.81 R	0.93	1.01 (0.83, 1.23)	N
MBL2	rs1800451 [16]	A	0.03	0.02	0.059 T	0.061	1.88 (0.96, 3.67)	P
MEFV	rs224204 [17]	T	0.49	0.47	0.36 D	0.38	1.09 (0.90, 1.33)	N
NLRP3^0^	rs3806265 [17]	C	0.34	0.35	0.46 D	0.54	0.94 (0.76. 1.15)	N
NOD2	rs1861759 [17]	C	0.39	0.37	0.26 D	0.64	1.05 (0.86, 1.28)	N
NRBF2-EGR2	rs10995450 [9]	T	0.24	0.24	0.96 R	0.98	0.998 (0.79, 1.25)	N
PRKCQ	rs4750316 [15]	C	0.17	0.18	0.33 R	0.42	0.90 (0.70, 1.17)	N
PSTPIP1^0^	rs4078354 [17]	T	0.33	0.36	0.13 A		0.85 (0.69, 1.05)	P
PTPN2	rs1893217 [6]	C	0.20	0.18	0.50 A	0.5	1.09 (0.85, 1.40)	N
	rs7234029 [2,6]	G	0.19	0.18	0.43 D	0.49	1.09 (0.85, 1.40)	N
	rs2542151 [6]	G	0.20	0.18	0.49 D	0.53	1.08 (0.85, 1.39)	N
PTPN22	rs2476601 [6,18]	A	0.10	0.07	0.006 A	0.006	1.62 (1.15, 2.30)	Y
RANTES (CCL5)	rs2107538 [19]	T	0.21	0.18	0.039 D	0.097	1.23 (0.96, 1.58)	P∞
	rs2280788 [19]	G	0.02	0.02	0.30 T	0.31	1.45 (0.71, 2.96)	N
SLITRK5*	rs1074044 [22]	C	0.44	0.44	0.81 D	0.9	1.01 (0.83, 1.23)	N
STAT4	rs8179673 [15]	C	0.29	0.24	0.012 D	0.024	1.29 (1.03, 1.61)	Y
	rs3821236 [6]	A	0.25	0.21	0.013 D	0.038	1.28 (1.01, 1.61)	Y
	rs7574865 [6,8,15]	T	0.29	0.24	0.0091 D	0.025	1.29 (1.03, 1.61)	Y
TNFAIP3	rs6920220 [8,15]	A	0.22	0.20	0.29 A	0.29	1.14 (0.90, 1.44)	N
	rs13207033 [15]	A	0.26	0.29	0.26 D	0.29	0.89 (0.71, 1.11)	N
TRAF1-C5	rs2900180 [15]	T	0.37	0.32	0.014 R	0.046	1.23 (1.00, 1.51)	Y
	rs3761847 [21]	A	0.42	0.40	0.32 A	0.32	1.11 (0.90, 1.36)	N
VTCN1	rs12046117 [22]	T	0.17	0.15	0.33 A	0.33	1.14 (0.87, 1.49)	N

* No gene attribution in original publication, closest gene by UCSC Genome Browser listed.

† Associated specifically with enthesitis related JIA in original report.

^0^ Associated specifically with psoriatic JIA in original report.

^§^ Model providing most significant P value: A = allelic, G = genotypic, T = Cochrane Armitage Trend Test (additive), D = dominant, R = recessive.

‡ Evidence for replication Y = Yes, P = Partial, N = No. See text for definitions. ? = potential for A/T allele reversal with respect to the prior report.

∞ Finding listed as P (partially replicated) since for this SNP, prior evidence was for a non-significant trend towards association in a direction consistent with our own.

We then performed a sensitivity analysis in which we included only cases (n = 200) and controls (n = 341) of European ancestry using the stringent definition of self-reported European ancestry of all four of the child’s grandparents. Non-European participants, along with participants for whom full grandparent data was not provided, were excluded. The outcome of this re-analysis is shown in Additional file 1: Table S1 and Additional file 1: Figure S1. In general, the results of this European-only analysis were not materially different to the full sample analysis (taking into account reduced statistical power resulting from a reduction of sample size), with one exception. *ATXN2* appeared influenced by ethnicity, with an opposite, non-significant, direction of effect in the European subgroup.

In conclusion, we have provided independent replication data for JIA susceptibility loci that have previously been identified using a generally limited number of international sample collections. We have confirmed association of JIA with SNPs close-by to *c12orf30*, *c3orf1*, *PTPN22*, *STAT4*, and *TRAF1-C5*; and we have provided further support for the association of SNPs close-by to *AFF3*, *CD226*, *MBL2*, *PSTPIP1*, and *CCL5*. A limitation of our study was our relatively small sample size; our full dataset had 80% power to detect an OR of 1.4 for an allele at 20% frequency in the population at an alpha of 0.05. Given that many of the published ORs were less than 1.4, and that a number of the SNPs analysed had minor allele frequencies less than 20%, we cannot, from our current data, *exclude* association of any of the SNPs examined. Our current sample size also precluded detailed subtype-specific association analyses. Large collaborative GWAS efforts would be beneficial in confirming the outstanding genes, and providing further novel insights into the breadth of genetic loci involved in JIA susceptibility.

## Competing interests

The authors declare that they have no competing interests.

## Authors’ contributions

JE and JM conceived, designed and led the study. RC performed the SNP genotyping. AP managed the study data. ALP advised and assisted with study design and collection of epidemiological data. RA and JA assisted with case study design, and JM, RA and JA assisted with recruitment through their paediatric rheumatology clinics. JE wrote the manuscript, and all authors participated in drafting the manuscript to the final version. All authors read and approved the final manuscript.

## Supplementary Material

Additional file 1 Data pertaining to SNPs removed from analysis during QC, and re-analysis of SNP association restricted to those of confirmed European descent. Click here for file
