# Supplementary material for: Independent replication analysis of genetic loci with previous evidence of association with juvenile idiopathic arthritis
Source: Pediatr Rheumatol Online J. 2013 Mar 18;11:12. doi: 10.1186/1546-0096-11-12 (PMC3606368; doi:10.1186/1546-0096-11-12)
Supplement: Additional file 1 — Data pertaining to SNPs removed from analysis during QC, and re-analysis of SNP association restricted to those of confirmed European descent. [file 1546-0096-11-12-S1.pdf]

Supplementary Results

Five SNPs failing QC were excluded from analysis.

The following SNPs were excluded due to poor raw data clustering and resultant low genotype call rate:

rs333 (CCR5)
rs5030737 (MBL2)
rs1800629 (TNFA)

The following SNPs were excluded because Hardy Weinberg Equilibrium $p < 0.01$ in controls:
rs1773560 (CD247)
rs17810546 (IL12A)

Supplementary Table 1: CLARITY SNP association results, restricted to those of European ancestry, as defined by ethnicity data obtained for all four of the child's grandparents. Case n = 200, control n = 341.

Gene	SNP	Minor allele	Case MAF	Control MAF	Best Test P[§]	Allelic	
						P	OR (95% CI)
ADAD1-IL2-IL21	rs17388568	A	0.30	0.28	0.44 T	0.44	1.11 (0.85, 1.46)
	rs13143866	A	0.27	0.26	0.23 R	0.78	1.04 (0.79, 1.38)
	rs6822844	T	0.15	0.13	0.31 R	0.43	1.16 (0.81, 1.65)
AFF3	rs1160542	G	0.49	0.45	0.20 R	0.23	1.16 (0.91, 1.49)
ANGPT1	rs1010824	T			FAILED QC		
ATXN2	rs653178	G	0.41	0.47	0.022 R	0.054	0.78 (0.61, 1.01)
BACH2	rs11755527	G	0.44	0.47	0.23 D	0.27	0.87 (0.68, 1.12)
C12orf30	rs17696736	G	0.53	0.48	0.031 D	0.099	1.23 (0.96, 1.58)
C3orf1	rs4688011	A	0.23	0.18	0.041 D	0.051	1.35 (1.00, 1.83)
CD14	rs2569190	A	0.49	0.50	0.57 R	0.74	0.96 (0.75, 1.23)
CD226	rs763361	T	0.54	0.47	0.051 T	0.051	1.28 (1.00, 1.64)
CLEC16A	rs6498169	G	0.36	0.35	0.62 R	0.85	1.03 (0.79, 1.33)
COG6	rs7993214	T	0.30	0.29	0.46 D	0.54	1.09 (0.83, 1.43)
CTLA4	rs3087243	A	0.46	0.48	0.34 R	0.60	0.94 (0.73, 1.20)
DCN1*	rs939898	G	0.24	0.19	0.044 D	0.055	1.34 (0.99, 1.81)
ERAP1	rs30187	T	0.38	0.36	0.44 D	0.67	1.06 (0.82, 1.37)
FHIT*	rs9311745	C	0.05	0.07	0.25 T	0.25	0.73 (0.42, 1.25)
HUNK*	rs2833547	T	0.29	0.28	0.88 D	0.90	1.02 (0.77, 1.34)
IL15	rs13139573	T	0.47	0.45	0.027 D	0.48	1.09 (0.85, 1.40)
IL23R	rs11209026	A	0.07	0.06	0.51 T	0.52	1.18 (0.72, 1.94)
	rs11465804	G	0.06	0.06	0.71 T	0.72	1.10 (0.65, 1.85)
IL2RA	rs12251307	T	0.10	0.12	0.25 A	0.25	0.79 (0.53, 1.18)
	rs706778	A	0.45	0.44	0.28 R	0.63	1.06 (0.83, 1.36)
	rs2104286	G	0.21	0.24	0.15 R	0.37	0.87 (0.64, 1.18)
IL7R	rs6897932	T	0.25	0.24	0.58 D	0.77	1.04 (0.78, 1.39)
JMJD1C-REEP3	rs6479891	T	0.17	0.17	0.35 R	0.80	0.96 (0.69, 1.33)
	rs12411988	C	0.16	0.17	0.47 D	0.63	0.92 (0.66, 1.29)
KIF5A	rs1678542	C	0.34	0.35	0.55 D	0.75	0.96 (0.74, 1.24)
LPP	rs1464510	T	0.44	0.48	0.026 D	0.19	0.85 (0.66, 1.08)
MBL2	rs1800451	A	0.03	0.01	0.051 T	0.053	2.31 (0.97, 5.54)

MEFV	rs224204	T	0.48	0.48	0.55 D	0.81	1.03 (0.80, 1.32)
NLRP3	rs3806265	C	0.31	0.36	0.048 D	0.12	0.81 (0.62. 1.06)
NOD2	rs1861759	C	0.39	0.38	0.51 D	0.84	1.03 (0.80, 1.32)
NRBF2-EGR2	rs10995450	T	0.20	0.21	0.54 T	0.55	0.91 (0.67, 1.24)
PRKCQ	rs4750316	C	0.16	0.18	0.33 R	0.45	0.88 (0.63, 1.23)
PSTPIP1	rs4078354	T	0.33	0.37	0.096 R	0.17	0.83 (0.64, 1.08)
PTPN2	rs1893217	C	0.21	0.20	0.51 R	0.53	1.10 (0.81, 1.50)
	rs7234029	G	0.21	0.18	0.14 A	0.14	1.26 (0.93, 1.72)
	rs2542151	G	0.22	0.20	0.45 D	0.47	1.12 (0.83, 1.52)
PTPN22	rs2476601	A	0.11	0.08	0.096 A	0.096	1.45 (0.93, 2.27)
RANTES (CCL5)	rs2107538	T	0.20	0.16	0.057 T	0.070	1.34 (0.98, 1.85)
	rs2280788	G	0.03	0.02	0.27 T	0.28	1.58 (0.69, 3.61)
SLITRK5*	rs1074044	C	0.47	0.45	0.47 T	0.47	1.10 (0.86, 1.40)
STAT4	rs8179673	C	0.28	0.22	0.015 D	0.021	1.40 (1.05, 1.85)
	rs3821236	A	0.23	0.19	0.038 D	0.077	1.31 (0.97, 1.78)
	rs7574865	T	0.28	0.22	0.014 D	0.019	1.40 (1.06, 1.86)
TNFAIP3	rs6920220	A	0.22	0.21	0.77 D	0.79	1.04 (0.77, 1.41)
	rs13207033	A	0.27	0.28	0.36 D	0.61	0.93 (0.71, 1.23)
TRAF1-C5	rs2900180	T	0.38	0.32	0.071 A	0.071	1.27 (0.98, 1.64)
	rs3761847	A	0.44	0.38	0.045 A	0.045	1.30 (1.01, 1.68)
VTCN1	rs12046117	T	0.15	0.14	0.42 A	0.42	1.15 (0.81, 1.64)

* No gene attribution in original publication, closest gene by UCSC Genome Browser listed

§ Model providing most significant P value: A = allelic, G = genotypic, T = Cochrane Armitage Trend Test (additive), D = dominant, R = recessive

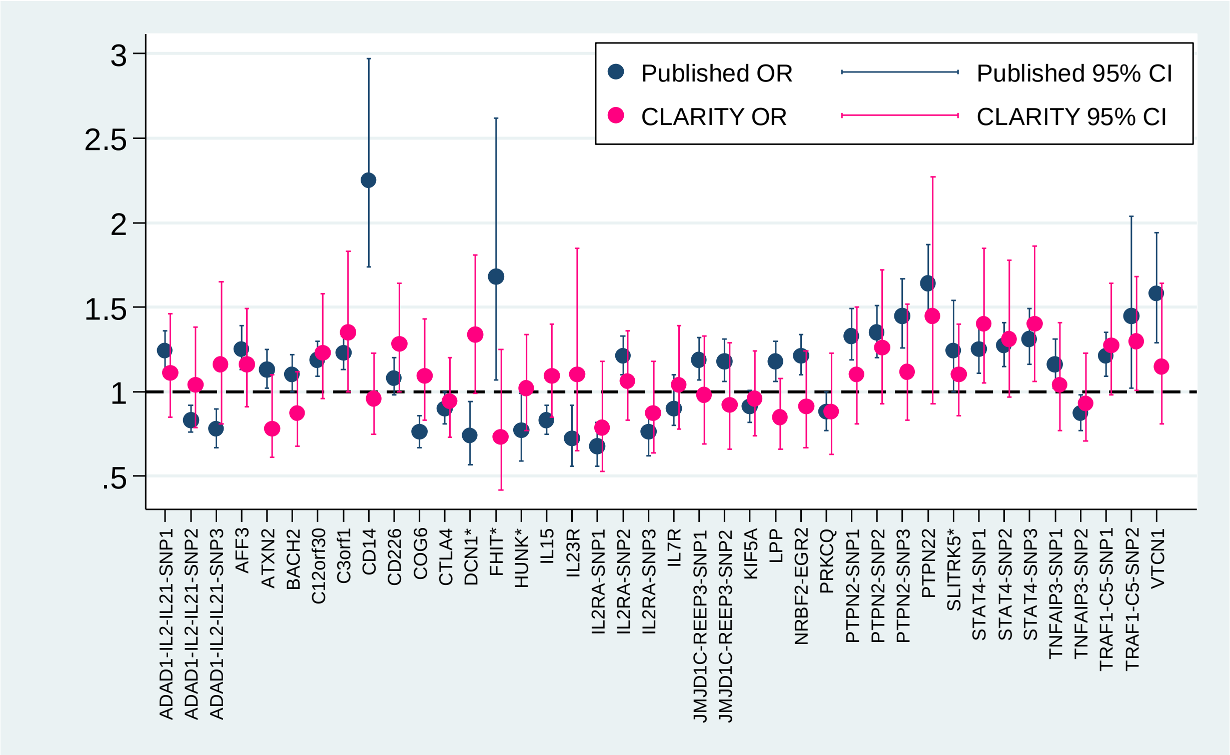

Supplementary Figure 1. Forest Plot comparing previously published odds ratios (ORs) with CLARITY ORs (European ancestry samples only) for genes examined via nearby SNPs.

Where more than one previously published OR was identified for a SNP, the OR based on the largest sample size was used. Full data is available in Supplementary Table 1. Some examined genes were excluded from this figure (see text for explanation). For genes with multiple SNPs (eg ADAD1-IL2-IL21) the SNPs are presented in the same order as listed in Supplementary Table 1.
* No gene attribution in original publication, closest gene by UCSC Genome Browser listed.
